# Importance of circulating adipocytokines in multiple myeloma: a systematic review and meta-analysis based on case-control studies

**DOI:** 10.1186/s12902-022-00939-2

**Published:** 2022-01-25

**Authors:** Rui Liu, Dandan Gao, Yang Lv, Meng Zhai, Aili He

**Affiliations:** 1 Department of Hematology, The Second Affiliated Hospital of Xi’an Jiaotong University, 157, 5th West Road, Xi’an, 710004 Shaanxi China; 2 National-Local Joint Engineering Research Center of Biodiagnostics & Biotherapy, The Second Affiliated Hospital of Xi’an Jiaotong University, Xi’an, 710004 Shaanxi China

**Keywords:** Leptin, Adiponectin, Resistin, Multiple myeloma, Meta-analysis

## Abstract

**Background:**

Adipocytes and their products, adipocytokines, play important roles in the generation and development of multiple myeloma (MM). Studies have demonstrated some adipocytokines to be associated with MM, although those results are controversial. Therefore, we conducted a meta-analysis to verify the association of adipocytokines with MM.

**Methods:**

We performed a systematic retrieval of literature published prior to 26 October 2021. Standardized mean difference (SMD) with a 95% confidence interval (CI) was calculated to evaluate pooled effects. Subgroup analysis and meta-regression analysis were conducted to detect sources of heterogeneity. Sensitivity analysis was performed to evaluate the stability of the study. Publication bias was assessed by funnel plots and Egger’s linear regression test.

**Results:**

Ten eligible studies with 1269 MM patients and 2158 controls were included. The pooled analyses indicated that circulating leptin levels of MM patients were significantly higher than control levels (SMD= 0.87, 95%CI: 0.33 to 1.41), while the circulating adiponectin levels in MM patients were significantly lower than controls with a pooled SMD of -0.49 (95%CI: -0.78 to -0.20). The difference of circulating resistin levels were not significant between MM patients and controls (SMD= -0.08, 95%CI: -0.55 to 0.39). Subgroup analysis and meta-regression analysis found that sample size, age, and sex were possible sources of heterogeneity. Sensitivity analysis demonstrated our pooled results to be stable.

**Conclusion:**

Decreased circulating adiponectin and increased leptin levels were associated with the occurrence and development of MM. Adiponectin and leptin may be potential biomarkers and therapeutic targets for MM.

**Supplementary Information:**

The online version contains supplementary material available at 10.1186/s12902-022-00939-2.

## Introduction

Multiple myeloma is a neoplastic plasma cell disease characterized by clonal proliferation of malignant mature B cells in bone marrow. Rapid proliferation of myeloma cells results in the accumulation of monoclonal proteins in the blood and urine. MM is the second most frequent hematological malignancy, accounting for 1.79% of all new cancer cases and 2.11% of all cancer deaths worldwide [1]. Older age, sex, African race, positive cancer history, and monoclonal gammopathy of undetermined significance (MGUS) are the recognized risk factors for MM [2]. A lifestyle-related factor considered to be an established risk factor for MM is obesity [3].

The bone marrow microenvironment plays an important role in the generation and progression of myeloma. The major component of BMME is the bone marrow adipose tissue (BMAT), which comprises 50-70% of BM volume and accounts for 5-10% of total fat [4]. It is well established that adipose tissue is not only an energy depot, but also an endocrine organ for the secretion of a number of adipocytokines: interleukin-6, tumor necrosis factor-alpha (TNF-α), adiponectin, leptin, resistin, and visfatin, which regulate energy metabolism, hematopoiesis, inflammation and tumorigenesis [5]. Of note, adipocytes corrupted by myeloma cells not only boost tumor growth but also safeguard cancer cells from chemotherapy-induced apoptosis. Further, adipocytokines derived from “reprogrammed” adipocytes can skew the balance between osteoblasts and osteoclasts resulting in the development of myeloma-associated bone disease [6–8].

Leptin is a 16-kDa peptide hormone, found at increased levels in overweight individuals, regulates energy balance and suppresses appetite via the hypothalamus. Leptin is a pro-inflammatory and pro-angiogenic factor that regulates cell proliferation and immune responsiveness via paracrine signaling [9]. Conversely, adiponectin is negatively associated with body mass index (BMI). Adiponectin is a pleiotropic cytokine with the capacity to sensitize cell to insulin, produce anti-inflammatory effects and exert anti-neoplastic activities [10]. Resistin, as its name implies, can lead to resistance to the action of insulin, impairment of energy homeostasis and the development of diabetes mellitus. Moreover, resistin is an inflammatory regulator that induces the overexpression of TNF-α, IL-6, IL-12, and monocyte chemotactic protein-1 (MCP-1) by activating the NF-κB signaling pathway [11]. Visfatin, an obesity-related adipocytokine, has roles in angiogenesis, anti-apoptosis, and inducing inflammation. Its intracellular form is nicotinamide phosphoribosyl transferase (NAMPT), which is involved in the synthesis of nicotinamide adenine dinucleotide (NAD) [12]. Visfatin can function as a pre-B cell colony enhancing factor (PBEF) for B cell development [13].

Yoon et al. reported that circulating adiponectin and leptin were significantly associated with risks of obesity-related cancers, such as endometrial cancer and breast cancer [14]. Furthermore, previous meta-analysis found that resistin and visfatin were significantly related to cancer risk [15, 16]. Although several epidemiological experiments examined the association among these adipocytokines and MM, results have been controversial and inconsistent, and are likely due to methodological and study population differences. Herein, we conducted a systematic review and meta-analysis that evaluated the role of adipocytokines in MM. The adipocytokines evaluated were leptin, adiponectin, resistin and visfatin.

## Materials and methods

We followed the Preferred Reporting Items for Systematic Reviews and Meta-Analyses (PRISMA) 2009 checklist [17]. This study has been registered on PROSPERO with the registration number CRD42021228394.

### Search strategy

Two investigators independently conducted a systematic literature retrieval in PubMed, Web of Science, and EMBASE to identify relevant studies prior to 26 October 2021. The search terms were as follows; “multiple myeloma” or “plasma cell neoplasm” combined with “adipokines” OR “adiponectin” OR “leptin” OR “resistin” OR “visfatin”. In addition, we scrutinized the references of relevant articles and reviews manually to add additional studies to the meta-analysis. The detailed search strategy is shown in Supplementary Methods.

### Inclusion and exclusion criteria

Publications were eligible if they met all of the following inclusion criteria: (1) case-control or cohort studies published as original articles, as well as other original articles containing relevant raw data; (2) studies evaluating associations among circulating adipocytokine levels and myeloma; (3) all patients were definitely diagnosed multiple myeloma; (4) sufficient data were available for the estimation of SMD with 95% CI.

The exclusion criteria were the following: (1) review papers, opinions, editorials, nonoriginal studies, interim analysis, pooled analysis, and studies published in multiple journals that were based on the same data; (2) conference abstracts that lacked baseline information and could not conduct methodological evaluations; (3) lack of the control group.

Two authors screened the relevant publications based on the above criteria. Discrepancies were resolved by discussion or consultation with another investigator.

### Data extraction

We performed data extraction based on Meta-analysis of Observational Studies in Epidemiology (MOOSE) checklist [18]. Variables were extracted as follows: last name of the first author, publication year, study design, country, sample size, control source, age, sex, BMI, diagnostic criteria, recruiting time of samples, test method, types of adipocytokines, circulating adipocytokines (mean, standard deviation), and unit. Information for eligible publications was collected independently by two reviewers, with re-evaluation by the third reviewer, if there was disagreement.

### Quality assessment

The quality of the selected studies was evaluated by two independent reviewers based on the Newcastle-Ottawa Scale (NOS) [19]. Disagreements were resolved by discussion. Total scores ranged from 0 to 9 based on three perspectives: selection, comparability and exposure. A study with a score greater than 6 was identified as a high-quality study. We used Grading of Recommendations, Assessment, Development, and Evaluations (GRADE) to assess the credibility of the current evidence [20].

### Statistical analysis

SMD with 95% CI was calculated based on the sample size, mean, and standard deviation of all eligible studies to assess the association between circulating concentration of adipocytokines and multiple myeloma. However, a few studies only reported median, quartiles, or maximum, and minimum. For those studies, we contacted the corresponding author for detailed information or estimated the sample mean and standard deviation from the available data by statistical methods [21, 22].

Heterogeneity was assessed by the chi-squared-based *Q* test and *I*^*2*^ statistics. Cut-offs of *P* < 0.10 and *I*^*2*^ > 50% were considered to be statistically significant heterogeneity. The pooled SMD was calculated by the fixed-effect model (FEM) if the heterogeneity was not significant. Otherwise, a random-effect model (REM) was calculated. Subgroup analysis and meta-regression analysis were conducted to detect underlying sources of heterogeneity. Sensitivity analysis was performed to confirm the effects reported by individual studies and to evaluate the stability of meta-analysis results.

Publication bias was assessed qualitatively by use of funnel plots and quantitatively by Egger’s linear regression test. Visual detection of asymmetry in funnel plots and a *P* < 0.05 indicated statistically significant publication bias. With publication bias, Trim and Fill analysis was used to assess the number of missing studies. The pooled results were re-evaluated with the addition of missing studies [23].

All data analysis was conducted with StataMP 14 software and Review Manager 5.4 software.

## Results

### Literature selection

**Fig. 1:**
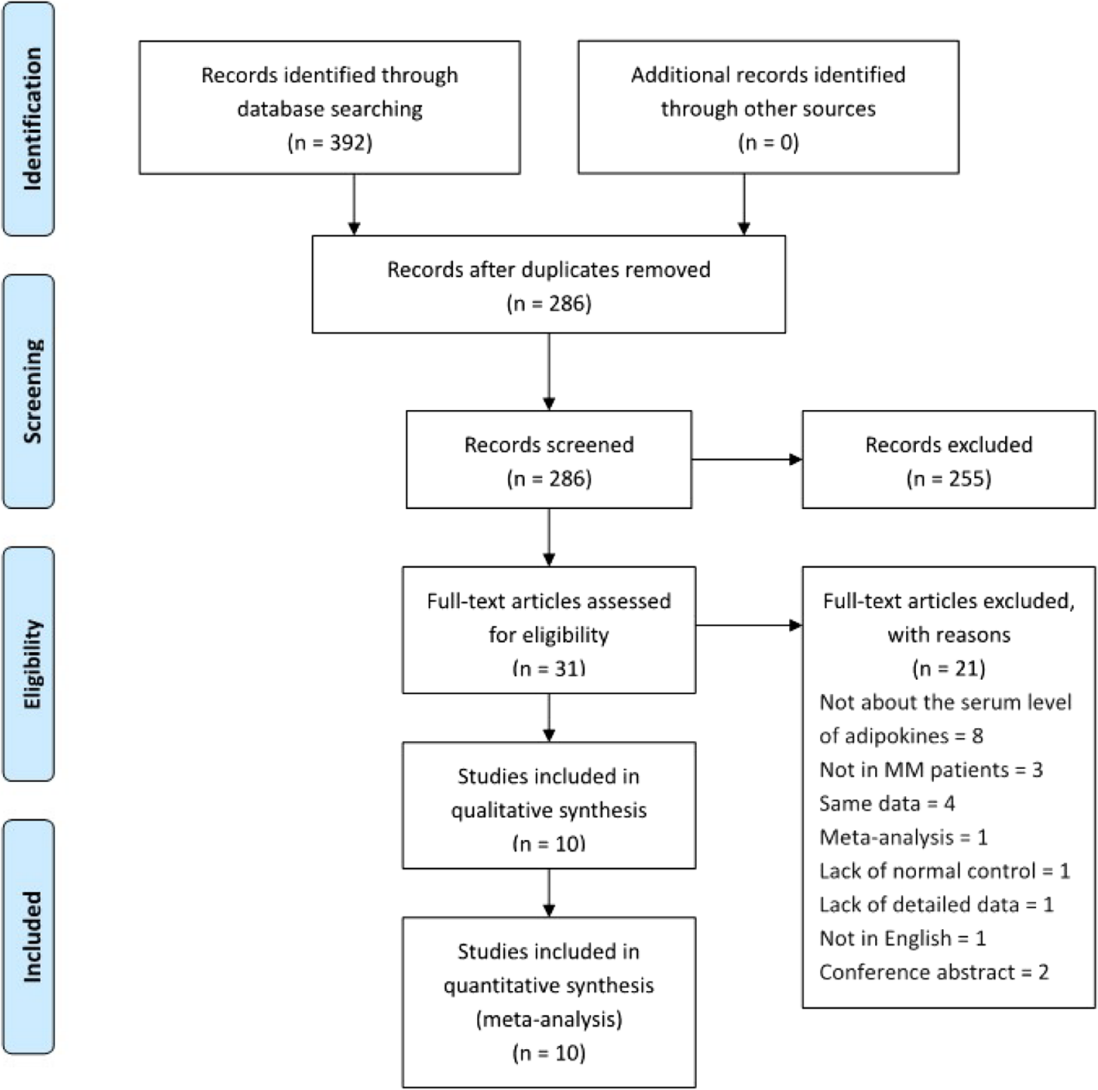
Flow diagram of the selection process for the meta-analysis

As shown in Fig. 1, the initial comprehensive search yielded 392 articles based on the search strategy (58 articles from PubMed, 143 articles from the Web of Science, 191 articles from EMBASE), of which 106 articles were excluded due to duplication. After scanning titles and abstracts, 255 articles were ruled out because of apparent irrelevance. More detailed selection resulted in 21 papers. Finally, 10 articles met the inclusion-exclusion criteria and were eligible for the meta-analysis [24–33].

### Study characteristics

The primary characteristics of all included studies are summarized in Table 1. A total of 1269 MM cases and 2158 age-matched controls were evaluated. Ten studies were conducted in six countries (two in Greece, three in USA, two in China, one in Turkey, one in Egypt, and one in Iraq). The age of most participants was between 60 and 65 years. No significant difference in age was observed between MM patients and controls. Nine of ten studies considered the impact of BMI on adipocytokine levels, and all individuals had a mean BMI well below the standard of WHO for obesity (BMI ≥30.0 kg/m^2^). All studies were case-control studies, with two nested case-control studies. A total of four different adipocytokines were evaluated. Seven studies included leptin [24–27, 30, 32], five studies included adiponectin [26, 27, 29, 30, 32], six studies included resistin [25, 26, 28, 30, 31, 33], and two studies included visfatin [30, 32]. Five of ten studies were scored above six and were considered as high-quality studies according to NOS. The detailed scoring process is shown in Table S1. According to GRADE assessment, all eligible studies were observative research without pluses and minuses, and were graded low. The detailed table is presented in Table S2.

**Table 1:** Characteristics of eligible studies in this meta-analysis

Author, year	country	recruiting year	Adipokines and test method	sample size, n		sex, n, male (female)		age, mean (SD)		BMI kg/m^**2**^ (Mean ± SD)		Diagnostic criteria	study design	source of control
				case	control	case	Control	Case	control	Case	control			
Alexandrakis 2004 [24]	Greece	NR	leptin (ELISA)	62	20	32(30)	sex-matched	68.3	age-matched	26.6±4.3	NR	Durie and Salmon criteria	case control	NR
Dalamaga 2009 [26]	Greece	18/1/2001 - 25/8/ 2007	leptin, resistin, adiponectin (ELISA)	73	73	42(31)	42(31)	67.1(5.9)	68.0(5.8)	27.7±4.0	26.2±4.2	Durie and Salmon criteria	case control	hospital
Hofmann 2012 [27]	USA	1993 - 2001	leptin, adiponectin (ELISA)	174	348	112(62)	224(124)	64.0	64.0	27.7±5.2	27.2±4.5	NR	nested case control	population
Hofmann 2016 [29]	USA	NR	adiponectin (ELISA)	627	1246	269(355)	538(708)	63.9(7.0)	63.9(7.0)	27.4±5.0	27.2±4.9	NR	nested case control	population
Liu 2020 [32]	China	NR	leptin, adiponectin, visfatin (ELISA)	39	20	24(15)	11(9)	63.4(8.4)	66.7(4.8)	NR	NR	International Myeloma Working Group	case control	hospital
Pamuk 2006 [25]	Turkey	NR	leptin, resistin (ELISA)	14	25	8(6)	15(10)	60.7(9.5)	54.5(12.0)	26.7±3.8	25.4±3.4	Durie and Salmon criteria	case control	hospital
Santo 2017 [31]	USA	NR	resistin (ELISA)	178	358	129(49)	258(100)	63.0(7.8)	63.0(7.8)	26.9±4.0	26.5±4.2	NR	nested case control	population
Yu 2016 [30]	China	NR	leptin, adiponectin, visfatin, resistin (ELISA)	28	28	15(13)	15(13)	56.0(10.0)	55.0(12.0)	22.8±3.1	23.0±2.8	International Myeloma Working Group	case control	hospital
Esheba 2014 [28]	Egypt	10/2011 - 10/2013	leptin, resistin (ELISA)	16	16	6(10)	6(10)	55.7(3.5)	55.5(4.0)	≤24.9 (non-obesity)		WHO diagnostic criteria	case control	Hospital
Salman 2020 [33]	Iraqi	10/2018 – 5/2019	Resistin (ELISA)	58	24	36(22)	15(9)	56.32	55.43	28.2	25.9	NR	Case control	Hospital

*NR* not reported

### Association of adipocytokines with MM

Detailed statistics for each adipocytokine are presented in Table 2. The statistics of ten studies were analyzed by REM to compare different circulating levels of adipocytokine in MM patients and controls.

**Table 2:** Circulating levels of leptin, adiponectin, resistin and visfatin in MM patients and controls

Author, year	Case			Control			Unit	***P*** value
	Mean	SD	N	Mean	SD	N		
**Circulating leptin levels**								
Alexandrakis 2004 [24]	17.96	20.39	62	4.18	0.96	20	ng/ml	<0.0001
Pamuk 2006 [25]	22.6	14.7	14	10.3	7.6	25	ng/ml	<0.01
Dalamaga 2009 [26]	27.5	17.6	73	21.9	9.5	73	ng/ml	0.02
Hofmann 2012 [27]	15.76	16.83	174	15.36	16.92	348	ng/ml	NS
Esheba 2014 [28]	5.82	1.43	16	2.16	0.72	16	ng/ml	<0.0001
Hu 2016	6.82	3.09	28	2.91	1.81	28	ng/ml	<0.01
Liu 2020 [32]	0.21	0.11	39	0.21	0.13	20	ng/ml	NS
**Circulating adiponectin levels**								
Dalamaga 2009 [26]	14.3	7.3	73	21.7	10.3	73	ng/ml	<0.0001
Hofmann 2012 [27]	10.42	6.98	174	11.68	7.84	348	μg/ml	NS
Hofmann 2016 [29]	12	6.3	624	13.1	6.8	1246	μg/ml	<0.05
Hu 2016	5.79	2.37	28	9.29	3.45	28	μg/ml	<0.01
Liu 2020 [32]	12.37	3.13	39	13.8	0.95	20	ng/ml	<0.05
**Circulating resistin levels**								
Pamuk 2006 [25]	3.3	3.3	14	1.97	0.6	25	ng/ml	NS
Dalamaga 2009 [26]	9.4	5	73	15.9	6.8	73	ng/ml	<0.0001
Esheba 2014 [28]	1.56	0.74	16	1.6	0.68	16	ng/ml	0.438
Hu 2016	8.98	6.41	28	9.48	6.18	28	ng/ml	0.091
Santo 2017 [31]	5.56	2.26	178	5.89	2.16	358	ng/ml	NS
Salma 2020 [33]	1.967	3.595	58	0.604	0.622	24	ng/ml	0.009
**Circulating visfatin levels**								
Yu 2016 [30]	8.35	5.06	28	7.74	4.79	28	ng/ml	0.819
Liu 2020 [32]	102.76	90.41	39	22.55	21.41	20	ng/ml	<0.05

*NS* not significant

**Fig. 2:**
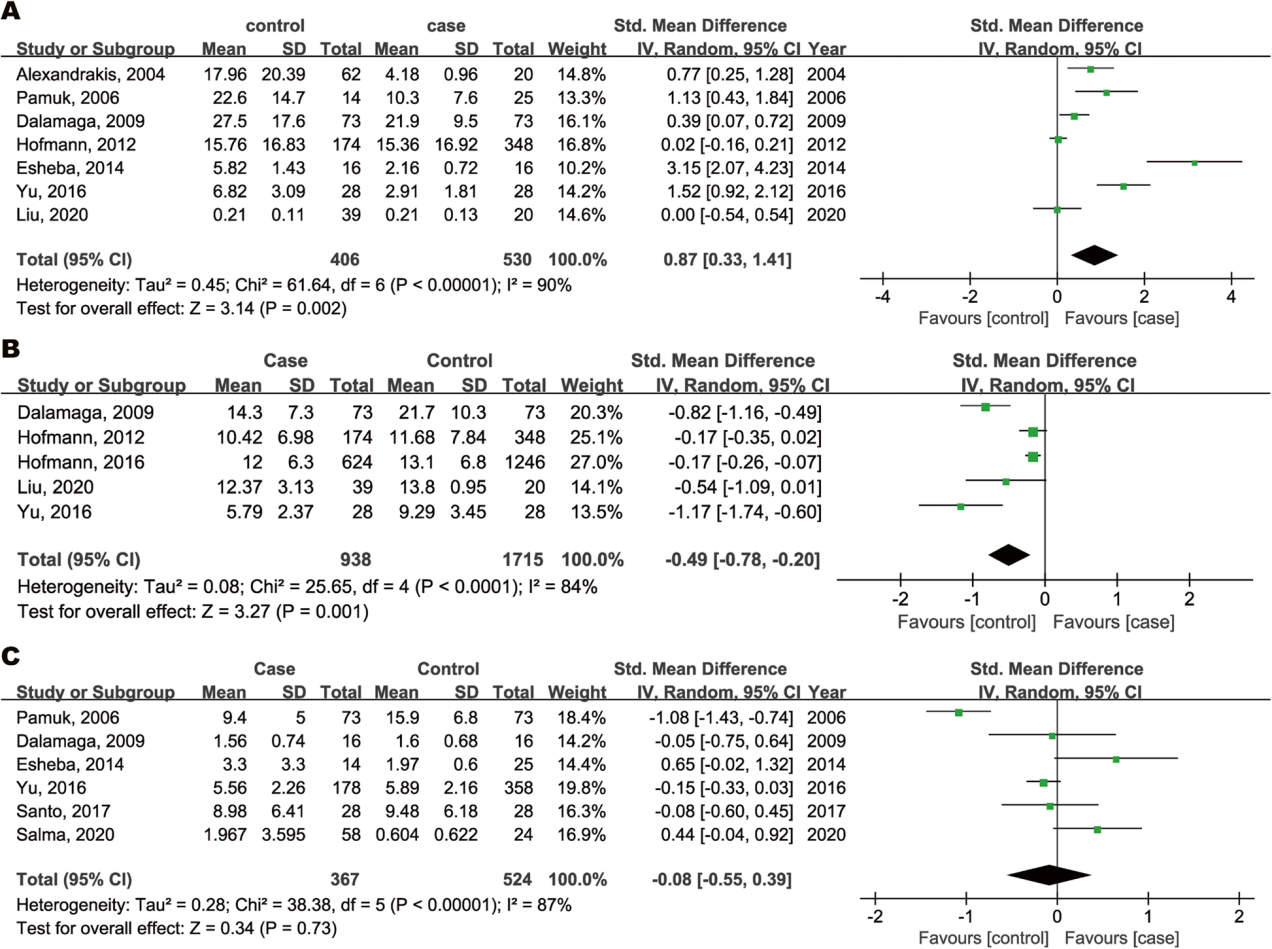
Forest plots of SMD with 95% CI of circulating leptin (**A**), adiponectin (**B**) and resistin (**C**) levels between MM patients and controls

Five out of seven studies found MM patients to have significantly increased circulating leptin concentrations compared to controls. As shown in Fig. 2A, a total of 406 MM patients and 530 controls were included in the pooled analysis. Pooled results showed a significantly higher level of leptin in patients with MM than in controls (SMD = 0.87, 95CI: 0.33 to 1.41, *z* = 3.14, *P* = 0.002). However, statistically significant heterogeneity was observed in these studies (*P* < 0.00001, *I*^*2*^ = 90% 95%CI: 0.8250 to 0.9458).

For adiponectin levels, significant reductions in four of five studies were found in patients with MM. 938 MM patients and 1715 controls were analyzed for adiponectin. Circulating adiponectin levels in MM patients were significantly lower than in controls with a pooled SMD of -0.49 (95%CI: -0.78 to -0.20, *z* = 3.27, *P* = 0.001; Fig. 2B). Similarly, significant heterogeneity was observed among studies (*P* < 0.0001, *I*^*2*^ = 84%, 95%CI: 0.6507 to 0.9304).

Two of six studies observed that MM patients had significantly altered circulating resistin level compared with controls. Pooled analysis showed that circulating resistin levels were not significantly different for 367 MM patients and 524 controls (SMD = -0.08, 95%CI: -0.55 to 0.39, *z* = 0.34, *P* = 0.73). Significant heterogeneity was found among these five studies (*P* < 0.0001, *I*^*2*^ = 87%, 95%CI: 0.7394 to 0.9349; Fig. 2C).

Studies of visfatin were limited by sample size, with diametrically opposed conclusions drawn. Those studies were not included in the pooled analysis.

### Subgroup analysis and meta-regression

**Fig. 3:**
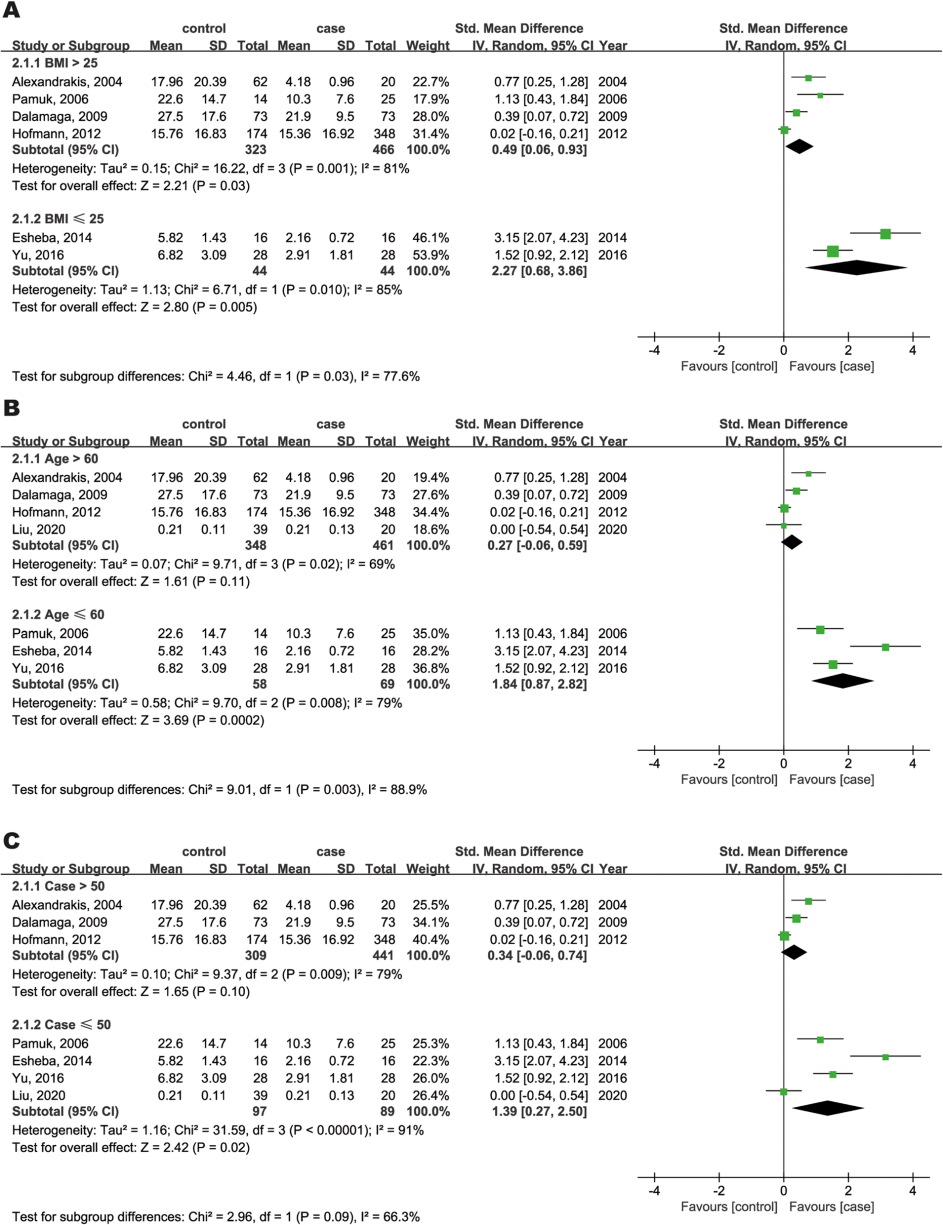
Forest plots of subgroup analysis by BMI (**A**), age (**B**) and case (**C**) in leptin

Subgroup analysis based on sample size, age, and BMI was performed to identify potential sources of leptin heterogeneity (Fig. 3). First, higher levels of circulating leptin were observed in overweight (SMD = 0.49, 95%CI: 0.06 to 0.93, Fig. 3A) and normal weight (SMD = 2.27, 95%CI: 0.68 to 3.86, Fig. 3A) MM patients than in controls. Second, compared with controls, MM patients under the age of 60 years had significantly higher levels of leptin (SMD = 1.84, 95%CI: 0.87 to 2.82, Fig. 3B), while those over 60 did not (SMD = 0.27, 95%CI: -0.06 to 0.59, Fig. 3B). Furthermore, in the subgroup of case size less than 50, MM patients had significantly higher leptin levels than controls (SMD = 1.39, 95%CI: 0.27 to 2.50, Fig. 3C), which was not true in the subgroup of case size more than 50 (SMD = 0.34, 95%CI: -0.06 to 0.74, Fig. 3C). Next, meta-regression analysis was performed to assess the impact of sample size, age, sex, region, BMI, study design, and NOS quality on SMD. Sex (*P* = 0.018) was found to be a significant contributing factor for between-study variance, while sample size (*P* = 0.262), age (*P* = 0.072), region (*P* = 0.070), BMI (*P* = 0.246), study design (*P* = 0.380), and NOS quality (*P* = 0.642) did not significantly affect study variance.

**Fig. 4:**
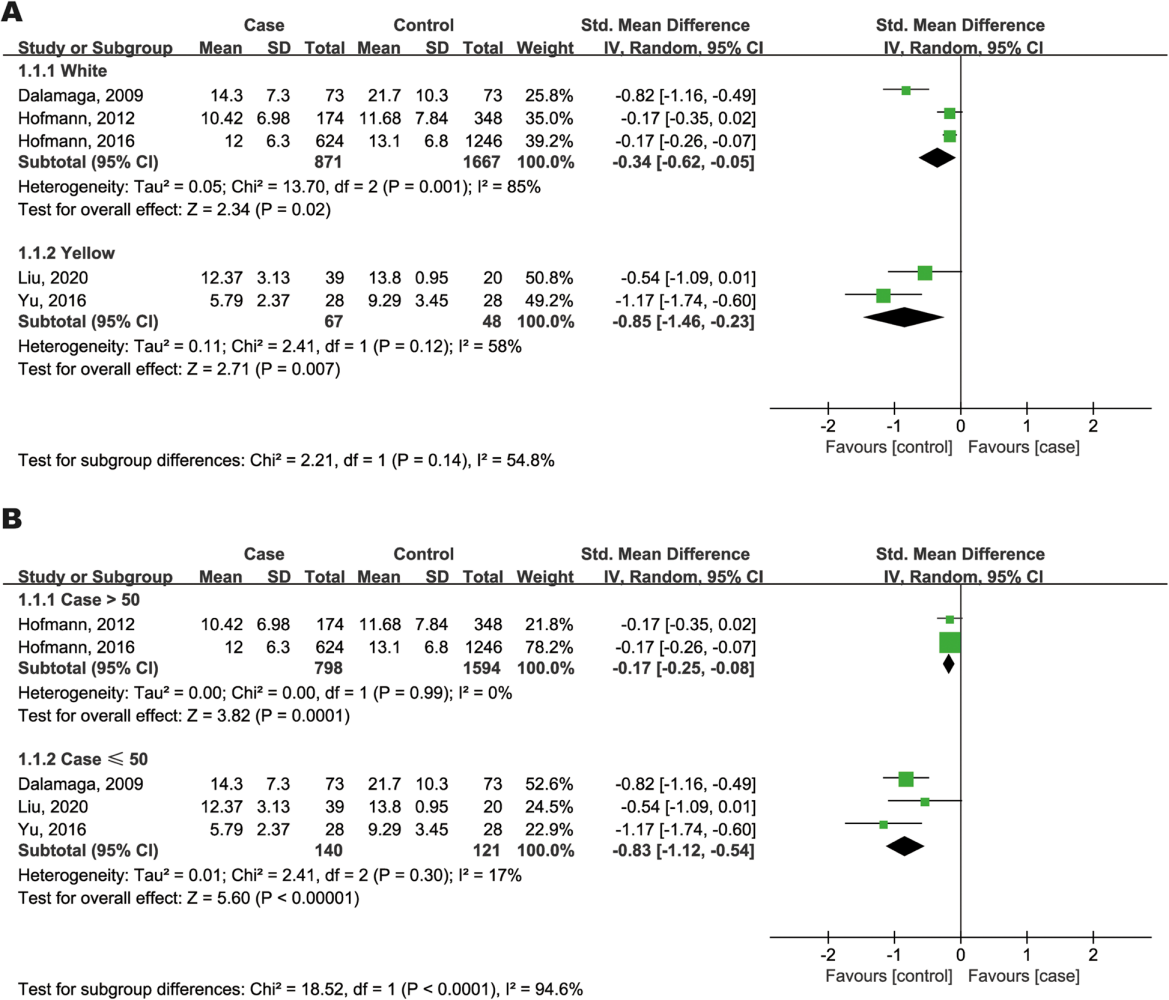
Forest plots of subgroup analysis by race (**A**) and age (**B**) in adiponectin

Subgroup analysis of adiponectin is summarized in Fig. 4. With regard to race, both Caucasian and Asian MM patients had significantly lower circulating adiponectin levels than controls (Fig. 4A). No matter the case size, decreased adiponectin levels were found in MM patients (SMD = -0.17, 95%CI: -0.25 to -0.08; SMD = -0.83, 95%CI = -1.12 to -0.54, Fig. 4B). Meta-regression analysis identified study design (*P* = 0.016) to be a significant contributing factor to heterogeneity, but sample size (*P* = 0.215), age (*P* = 0.440), region (*P* = 0.094), BMI (*P* = 0.197), sex (*P* = 0.919) and NOS quality (*P* = 0.665) were not.

### Sensitivity analysis

**Fig. 5:**
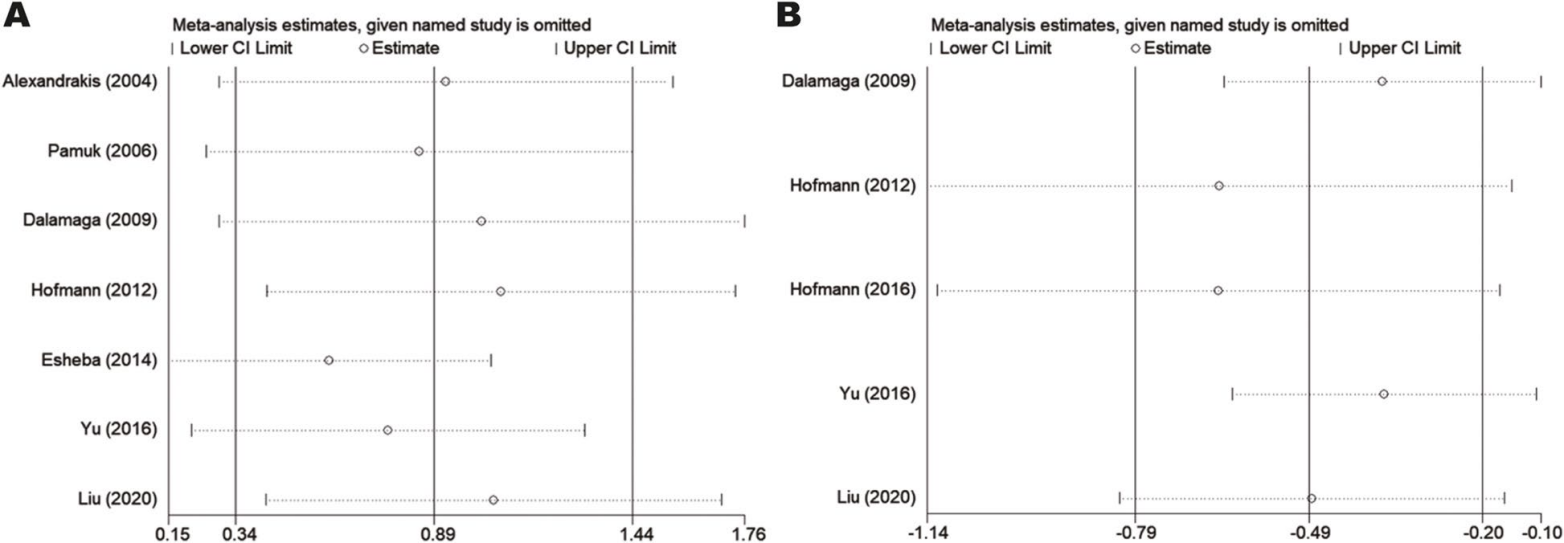
The pooled SMD and 95%CI of eligible studies of leptin and adiponectin through sensitivity analysis

Studies were individually excluded to evaluate the robustness of the results and the influence of each study on pooled SMD (Fig. 5A and B; Tables S3 and S4). The omission of any particular study did not appreciably change combined SMD. Estimates in each case were well within the confidence limits of the overall estimates, which implied the stability of results.

### Publication bias

For adiponectin there were only five eligible studies, and thus, Egger’s regression test rather than funnel plot was used to assess publication bias. No evidence of publication bias was found (*P* = 0.061). For leptin, evident asymmetry was observed in the funnel plot (Fig. S1), with an Egger’s test result (*P* = 0.01), indicating the significant publication bias. Therefore, Trim and Fill analysis was used to evaluate the effect of publication bias on pooled results. Analysis showed the imputed pooled results were identical to original results (SMD = 0.890, 95%CI: 0.337 to 1.442). No missing studies were added to the filled funnel plot (Fig. S2).

## Discussion

MM is an incurable neoplasm. Even though overall survival for MM patients has increased, patients' medical needs remain unmet. It is therefore important to explore underlying oncogenic mechanisms and novel predictive biomarkers that will allow the individualized cancer therapies for specific patients. Obesity has been well established as a risk factor for hypertension, cardiovascular disease, and type-2 diabetes mellitus. Accumulating epidemiological studies have demonstrated obesity to be a risk factor for cancers, including MM [34]. Obesity-related oncogenic mechanisms are involved in two major aspects: insulin resistance and chronic inflammation [35, 36], which are tightly associated with the secretion of adipocytokines.

Previous meta-analysis of a number of cancer patients demonstrated adiponectin and leptin to be associated with cancer generation. However, that analysis included only two MM studies [14, 37]. Moreover, the results of studies conducted to explore the associations among MM and adipocytokines have been inconsistent and limited. Therefore, we performed this meta-analysis to evaluate the associations of circulating levels of adiponectin, leptin, resistin, and visfatin with MM. The results suggested that increased circulating levels of leptin and decreased circulating levels of adiponectin were associated with a higher risk of MM. Circulating resistin levels did not differ between MM patients and controls. Due to the limited number of eligible studies, pooled analysis of visfatin was not conducted. Sensitivity analysis by sequential omission of individual studies produced statistically consistent results, which indicated the robustness of our meta-analysis. Moreover, adiponectin and leptin results are consistent with previous meta-analyses of renal cell carcinoma, breast, and colorectal cancer, which further supports the credibility of our results [14, 38–40]. In myeloma-associated bone disease, reprogrammed adipocytes increase leptin secretion while decrease adiponectin secretion to intensify osteoclastogenesis and to retard osteoblastogenesis, which is consistent with our pooled results [6]. These findings suggest that adipocytokine dysregulation affects bone marrow microenvironment and that the reprogrammed profile of adipocytokines may serve as novel promising indicator for myeloma-associated bone disease.

Circulating levels of adiponectin and leptin are in accordance with their cancer biological function. Leptin is an oncogenic factor due to its pro-inflammatory and pro-angiogenic effects. Yu et al. [41] demonstrated adipocyte-derived leptin to promote self-renewal of breast cancer stem cells and to induce chemo-resistance via the JAK/STAT3/CPT1B axis. Moreover, leptin is involved in the PI3K/AKT/lysyl hydroxylase signaling pathway, which facilitates breast cancer metastasis [42]. For MM, leptin plays a significant role in drug resistance by activating the JAK/STAT and PI3K pathways, enhancing proliferation and autophagy [30, 43].

Adiponectin has been identified as a “friendly” cytokine owing to its anti-tumor activity in breast cancer and colorectal cancer [44]. Several studies have demonstrated its tumor-suppressive activity in MM by inducing apoptosis [45]. Further, adiponectin can alleviate myeloma bone disease by inhibiting the differentiation and maturation of osteoclasts by activation of the mTOR pathway [32].

Resistin may play an oncogenic role in MM by serving as an inflammatory regulator. Previous pooled analysis demonstrated increased resistin circulating levels to be associated with a higher risk for colorectal cancer [46]. For breast cancer, resistin is involved in epithelial-mesenchymal transition and stemness, which are critical to metastasis and tumorigenesis via NF-κB/STAT3 pathways [47]. For MM, resistin can induce multidrug tolerance (melphalan, bortezomib, and carfilzomib) through NF-κB and PI3K/Akt pathways. Further, resistin can accelerate drug expulsion, epigenetically, via the DNA methyltransferases DNMT/ATP-binding cassette (ABC) transporter axis [48].

Visfatin is a significant adipocytokine due to its NAMPT activity, which is essential for the synthesis of NAD. Visfatin acts as an oncogenic factor by promoting proliferation, metastasis, angiogenesis, and drug resistance [49] in various solid tumors, including breast [50, 51], colorectal [52], and ovarian cancer [53]. Although studies in MM are limited, visfatin has been reported to be involved in myeloma bone disease and myeloma cell growth. Further, the depletion of intracellular NAD^+^ sensitizes bortezomib-induced cytotoxicity, indicating that visfatin could be a biomarker or therapeutic target for MM [54–56].

Heterogeneity was found in our pooled analyses. Subgroup analysis and meta-regression were used to identify the source of heterogeneity. Sample size, age, and sex were found to be possible sources of the heterogeneity for leptin. Study design might be the cause of heterogeneity for adiponectin. Furthermore, heterogeneity may be accounted for by the following clinical differences in MM studies. First, there may be differences in the demographic features and genetic background of Asian and Caucasian populations. Second, myeloma types, different stages, risk stratification, and tumor heterogeneity may be responsible. Third, non-myeloma related adipocytokine alterations such as BMI, past medical history, hormone receptor expression, menopausal status, and emerging endocrine diseases may have influenced the results. Another possible reason for heterogeneity may be that levels of adipocytokines vary by time of day, and a single blood sample measurement might be inadequate. Several methodological discrepancies were also observed. To avoid the effect of confounding factors, results of included research were adjusted for covariates such as sex and age, while few were adjusted for BMI. Furthermore, the source of controls was from hospital in eight of ten studies, but only three studies reported the strict exclusion criteria (such as obesity, diabetes, and hyperlipidemia). Thus, we suggest that future studies focused on adipocytokine should adjust for BMI, or make strict inclusion and exclusion criteria to avoid not cancer-related impact on adipocytokine levels.

There are several limitations in this meta-analysis. First, all included studies were case-control, with inherent selection, information, and confounding bias. Second, data regarding myeloma types, cancer stage, risk stratification, past medical history, and lifestyle was not available. Third, the sample sizes of few eligible studies are small.

In conclusion, results herein suggest that low circulating levels of adiponectin and high circulating levels of leptin are associated with an increased risk for MM, indicating that adiponectin and leptin may be potential biomarkers.

## Supplementary Information

**Additional file 1: Table S1.** Newcastle-Ottawa scale for the assessment of included studies. **Table S2.** Using GARDE to assess the credibility of the current evidence. **Table S3.** The pooled SMD and 95%CI of the eligible studies of leptin through sensitivity analysis. **Table S4.** The pooled SMD and 95%CI of the eligible studies of adiponectin through sensitivity analysis. **Figure S1.** Funnel plot for publication bias of the association between serum leptin levels and myeloma. Evident asymmetry was observed in the funnel plot, and the Egger’s test result was *P*=0.013, which indicates the existence of significant publication bias. **Figure S2.** Filled Funnel plot of the association between serum leptin levels and myeloma by the use of Trim and Fill method. Trim and Fill analysis was used to evaluate the effect of publication bias on the pooled result. The analysis showed the imputed pooled result was identical to our original result (SMD= 0.890, 95%CI: 0.337 to 1.442). No missing studies were added to the filled funnel plot.

## Data Availability

All data analyzed during this study are included in this published article.

## Abbreviations

BMAT: Bone marrow adipose tissue
BMI: Body mass index
CI: Confidence interval
ELISA: Enzyme-linked immunosorbent assay
FEM: Fixed-effect model
MGUS: Monoclonal gammopathy of undetermined significance
MM: Multiple myeloma
REM: Random-effect model
SMD: Standardized mean difference
TNF: Tumor necrosis factor
