# Supplementary material for: Importance of circulating adipocytokines in multiple myeloma: a systematic review and meta-analysis based on case-control studies
Source: BMC Endocr Disord. 2022 Jan 25;22:29. doi: 10.1186/s12902-022-00939-2 (PMC8787905; doi:10.1186/s12902-022-00939-2)
Supplement: Supplementary file 1 — Additional file 1: Table S1. Newcastle-Ottawa scale for the assessment of included studies. Table S2. Using GARDE to assess the credibility of the current evidence. Table S3. The pooled SMD and 95%CI of the eligible studies of leptin through sensitivity analysis. Table S4. The pooled SMD and 95%CI of the eligible studies of adiponectin through sensitivity analysis. Figure S1. Funnel plot for publication bias of the association between serum leptin levels and myeloma. Evident asymmetry was observed in the funnel plot, and the Egger’s test result was P=0.013, which indicates the existence of significant publication bias. Figure S2. Filled Funnel plot of the association between serum leptin levels and myeloma by the use of Trim and Fill method. Trim and Fill analysis was used to evaluate the effect of publication bias on the pooled result. The analysis showed the imputed pooled result was identical to our original result (SMD= 0.890, 95%CI: 0.337 to 1.442). No missing studies were added to the filled funnel plot. [file 12902_2022_939_MOESM1_ESM.docx]

Supplementary Material

# Supplementary Tables

**Table S1. Newcastle-Ottawa scale for the assessment of included studies**

| NEWCASTLE-OTTAWA QUALITY ASSESSMENT SCALE FOR CASE CONTROL STUDIES | | | | | | | | | | |
| --- | --- | --- | --- | --- | --- | --- | --- | --- | --- | --- |
| quality assessment criteria | Alexandrakis, 2004 | Dalamaga, 2009 | Hofmann, 2012 | Hofmann, 2016 | Liu, 2020 | Pamuk, 2006 | Santo, 2017 | Yu, 2016 | Esheba, 2014 | Salman, 2020 |
| adequate case definition | * | * | * | * | * | * | * | * | * | - |
| representativeness of cases | * | * | - | * | - | - | - | * | - | - |
| selection of controls | - | - | * | * | - | - | * | - | - | - |
| definition of controls | - | * | * | * | * | * | * | * | * | * |
| Comparability | ** | ** | ** | ** | * | * | ** | ** | ** | ** |
| ascertainment of exposure | - | * | * | - | - | * | * | - | - | - |
| same method of ascertainment for cases and controls | * | * | * | * | * | * | * | * | * | * |
| non-response rate | * | * | * | * | * | * | * | * | * | * |
| total scores | 6 | 8 | 8 | 8 | 5 | 6 | 8 | 7 | 6 | 5 |

(* = one score)

**Table S2. Using GARDE to assess the credibility of the current evidence**

| *Grading of Recommendations, Assessment, Development, and Evaluations (GRADE)* | | | | | | | | | | |
| --- | --- | --- | --- | --- | --- | --- | --- | --- | --- | --- |
| quality assessment criteria | Alexandrakis, 2004 | Dalamaga, 2009 | Hofmann, 2012 | Hofmann, 2016 | Liu, 2020 | Pamuk, 2006 | Santo, 2017 | Yu, 2016 | Esheba, 2014 | Salman, 2020 |
| Type | Observational study | Observational study | Observational study | Observational study | Observational study | Observational study | Observational study | Observational study | Observational study | Observational study |
| Study quality | 0 | 0 | 0 | 0 | 0 | 0 | 0 | 0 | 0 | 0 |
| Inconsistency | 0 | 0 | 0 | 0 | 0 | 0 | 0 | 0 | 0 | 0 |
| Uncertainty about directness | 0 | 0 | 0 | 0 | 0 | 0 | 0 | 0 | 0 | 0 |
| Imprecise or sparse data | 0 | 0 | 0 | 0 | 0 | 0 | 0 | 0 | 0 | 0 |
| High probability of reporting bias | 0 | 0 | 0 | 0 | 0 | 0 | 0 | 0 | 0 | 0 |
| Strong evidence of association | 0 | 0 | 0 | 0 | 0 | 0 | 0 | 0 | 0 | 0 |
| dose response gradient | 0 | 0 | 0 | 0 | 0 | 0 | 0 | 0 | 0 | 0 |
| All plausible confounders would have reduced the effect | 0 | 0 | 0 | 0 | 0 | 0 | 0 | 0 | 0 | 0 |
| Total | Low | Low | Low | Low | Low | Low | Low | Low | Low | Low |

**Table S3. The pooled SMD and 95%CI of the eligible studies of leptin through sensitivity analysis**

| study omitted | estimate | 95%CI |
| --- | --- | --- |
| Alexandrakis (2004) | 0.92278254 | 0.29040846 to 1.5551566 |
| Pamuk (2006) | 0.84933496 | 0.2543951 to 1.4442749 |
| Dalamaga (2009) | 1.0235088 | 0.29025909 to 1.7567585 |
| Hofmann (2012) | 1.0773054 | 0.42432958 to 1.7302812 |
| Esheba (2014) | 0.59805411 | 0.14778338 to 1.0483248 |
| Yu (2016) | 0.76064521 | 0.21181604 to 1.3094743 |
| Liu (2020) | 1.0567106 | 0.42011878 to 1.6933024 |
| Combined | 0.8895856 | 0.33691916 to 1.442252 |

**Table S4. The pooled SMD and 95%CI of the eligible studies of adiponectin through sensitivity analysis**

| study omitted | Estimate | 95%CI |
| --- | --- | --- |
| Dalamaga (2009) | -0.3681337 | -0.6375417 to -0.09872574 |
| Hofmann (2012) | -0.6452873 | -1.1422777 to -0.14829698 |
| Hofmann (2016) | -0.6466029 | -1.1243237 to -0.16888212 |
| Yu (2016) | -0.3648286 | -0.6231742 to -0.10648293 |
| Liu (2020) | -0.4884212 | -0.8153647 to -0.16147758 |
| Combined | -0.4932682 | -0.7886094 to -0.19792686 |

# Supplementary Figures

**
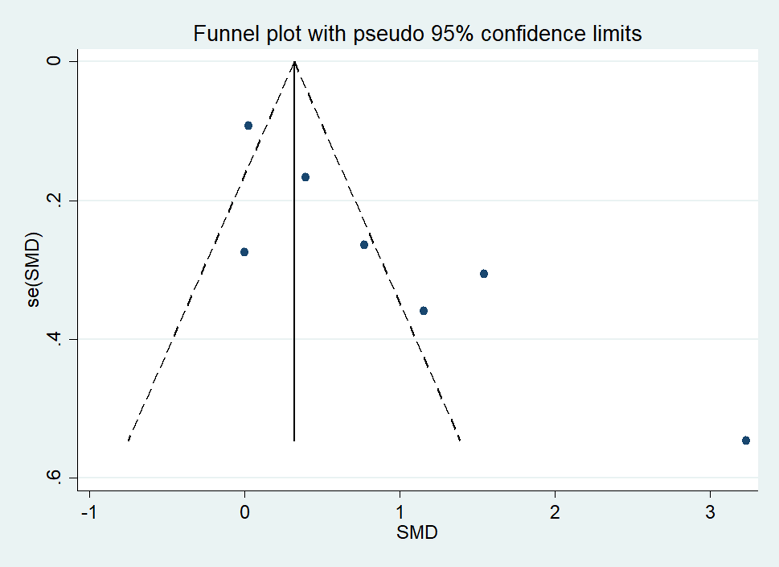
**

**Figure S1. Funnel plot for publication bias of the association between serum leptin levels and myeloma.** Evident asymmetry was observed in the funnel plot, and the Egger’s test result was *P*=0.013, which indicates the existence of significant publication bias.

**
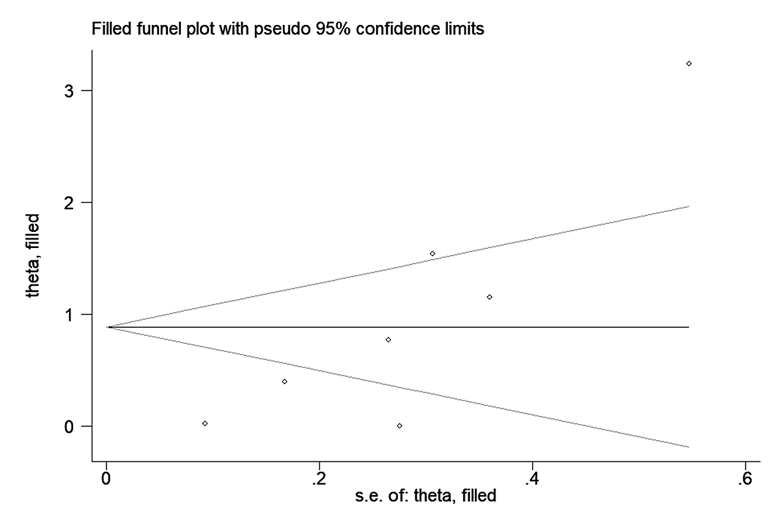
**

**Figure S2. Filled Funnel plot of the association between serum leptin levels and myeloma by the use of Trim and Fill method.** Trim and Fill analysis was used to evaluate the effect of publication bias on the pooled result. The analysis showed the imputed pooled result was identical to our original result (SMD= 0.890, 95%CI: 0.337 to 1.442). No missing studies were added to the filled funnel plot.

**3. Supplementary Methods**

*Search strategy in PubMed*

Search: **((plasma cell neoplasm) OR (multiple myeloma)) AND (((((adiponectin) OR (leptin)) OR (resistin)) OR (visfatin)) OR (adipocytokine))**

("neoplasms, plasma cell"[MeSH Terms] OR ("neoplasms"[All Fields] AND "plasma"[All Fields] AND "cell"[All Fields]) OR "plasma cell neoplasms"[All Fields] OR ("plasma"[All Fields] AND "cell"[All Fields] AND "neoplasm"[All Fields]) OR "plasma cell neoplasm"[All Fields] OR "multiple myeloma"[MeSH Terms] OR ("multiple"[All Fields] AND "myeloma"[All Fields]) OR "multiple myeloma"[All Fields] OR ("plasma"[All Fields] AND "cell"[All Fields] AND "neoplasm"[All Fields]) OR ("multiple myeloma"[MeSH Terms] OR ("multiple"[All Fields] AND "myeloma"[All Fields]) OR "multiple myeloma"[All Fields])) AND ("adiponectin"[MeSH Terms] OR "adiponectin"[All Fields] OR "adiponectin s"[All Fields] OR "adiponectine"[All Fields] OR "adiponectins"[All Fields] OR ("leptin"[MeSH Terms] OR "leptin"[All Fields] OR "leptins"[All Fields] OR "leptin s"[All Fields] OR "leptine"[All Fields] OR "leptines"[All Fields]) OR ("resistin"[MeSH Terms] OR "resistin"[All Fields]) OR ("nicotinamide phosphoribosyltransferase"[MeSH Terms] OR ("nicotinamide"[All Fields] AND "phosphoribosyltransferase"[All Fields]) OR "nicotinamide phosphoribosyltransferase"[All Fields] OR "visfatin"[All Fields]) OR ("adipokines"[MeSH Terms] OR "adipokines"[All Fields] OR "adipocytokine"[All Fields] OR "adipocytokines"[All Fields]))

**Translations**

**plasma cell neoplasm:** "neoplasms, plasma cell"[MeSH Terms] OR ("neoplasms"[All Fields] AND "plasma"[All Fields] AND "cell"[All Fields]) OR "plasma cell neoplasms"[All Fields] OR ("plasma"[All Fields] AND "cell"[All Fields] AND "neoplasm"[All Fields]) OR "plasma cell neoplasm"[All Fields] OR "multiple myeloma"[MeSH Terms] OR ("multiple"[All Fields] AND "myeloma"[All Fields]) OR "multiple myeloma"[All Fields] OR ("plasma"[All Fields] AND "cell"[All Fields] AND "neoplasm"[All Fields])

**multiple myeloma:** "multiple myeloma"[MeSH Terms] OR ("multiple"[All Fields] AND "myeloma"[All Fields]) OR "multiple myeloma"[All Fields]

**adiponectin:** "adiponectin"[MeSH Terms] OR "adiponectin"[All Fields] OR "adiponectin's"[All Fields] OR "adiponectine"[All Fields] OR "adiponectins"[All Fields]

**leptin:** "leptin"[MeSH Terms] OR "leptin"[All Fields] OR "leptins"[All Fields] OR "leptin's"[All Fields] OR "leptine"[All Fields] OR "leptines"[All Fields]

**resistin:** "resistin"[MeSH Terms] OR "resistin"[All Fields]

**visfatin:** "nicotinamide phosphoribosyltransferase"[MeSH Terms] OR ("nicotinamide"[All Fields] AND "phosphoribosyltransferase"[All Fields]) OR "nicotinamide phosphoribosyltransferase"[All Fields] OR "visfatin"[All Fields]

**adipocytokine:** "adipokines"[MeSH Terms] OR "adipokines"[All Fields] OR "adipocytokine"[All Fields] OR "adipocytokines"[All Fields]
